# Low-cost open-source camera view splitter (quadscope) for flow diagnostics

**DOI:** 10.1016/j.ohx.2025.e00723

**Published:** 2025-11-24

**Authors:** Abinash Sahoo, Ryan D. DeBoskey, Venkateswaran Narayanaswamy

**Affiliations:** Department of Mechanical and Aerospace Engineering, NC State University, Raleigh, NC 27695, USA

**Keywords:** View splitter, Quadscope, Image multiplier, Pyrometry, 3D printing, DIY

## Abstract

Camera view splitters use optical engineering to cast multiple images of a single target onto a single camera. For experiments in fluid dynamics, the ability to perform multivariate and multidimensional measurements is key to effectively studying complex thermofluidic phenomena. Generating multiple target views onto a single camera is a good way to reduce system costs by using fewer cameras. Currently, view splitting requires the purchase of expensive commercial lenses or the in-house design and fabrication of custom components, hindering the pace of research and development. Here, we provide an open-source design for a low-cost, general-purpose view splitter, providing up to four views per camera. All custom parts for the assembly are provided, which are designed for easy integration with standard optical components. Additional files are included for different view splitter orientations (designed for tomography) and the optional integration with spectral filtering. Characterization experiments were performed at multiple object distances to determine changes in image mapping quality, parallax angles, pixel density, and field-of-view (FOV). Validation is performed on a non-premixed methane/air flame, demonstrating the ability of the view splitter to perform simultaneous high-speed imaging and quantitative measurement in a practical system. This assembly has the potential to democratize access to advanced imaging capability and accelerate progress in the broader diagnostics community.

## Graphical abstract

**Figure:**
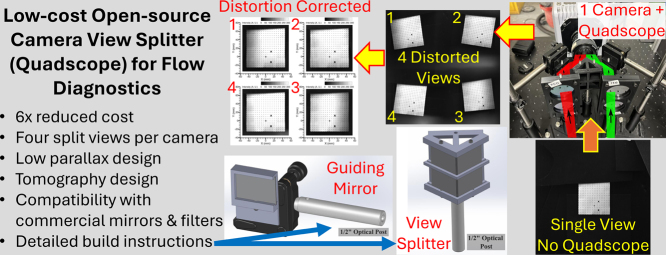

## Specifications table

**Table:** 

Hardware name	Low-cost camera view splitter (quadscope)

Subject area	General

Hardware type	Imaging tools

Closest commercial analog	•LaVision Image Doubler, Part #1108765/1108766, ∼$8,000 •LaVision Image Doubler for Pyrometry, Part #1108532, ∼$11,000 LaVision

Open source license	CC BY 4.0

Cost of hardware	•$1,208 •$2,678 (including spectral filters)

Source file repository	https://doi.org/10.17632/3pmyf5vghd.1

	

## 1 Hardware in context

Advanced flow diagnostics often necessitate multivariate or multidimensional measurement of flow properties to elucidate complex thermofluidic behaviors. Toward this end, simultaneous imaging of multiple parameters (pressure, temperature, velocity, and species concentrations), multi-color pyrometry, and tomographic imaging frequently employ multiple cameras, which can elevate system cost [1], [2], [3]. As a solution, a single camera can be used with an appropriate optical configuration to generate multiple views of the same target. This work focuses on the quadscope view splitter design, which is general-purpose for both view multiplication and tomographic applications, providing up to four views per camera.

View multiplication is accomplished through optical engineering, relying mainly on the use of beam-splitting optics and mirrors to route the incoming light into multiple views. Different configurations of optical components are often used, each with distinct advantages in cost and accuracy [4]. For view multiplication, the primary objective is to reduce optical distortions to maximize parity between images. The main source of error is the parallax, which occurs due to differences in the orientation of optical path lines between channels. Using beam-splitting optics and multiple cameras, view multiplication can be performed with perfect parity, completely removing any parallax or path length errors [5]. Unfortunately, this methodology requires a separate camera and lens for each split view, quickly elevating system cost and rendering this option unfeasible for many researchers. An attractive alternative is to direct the split images onto a single camera, partitioning the camera sensor for each image view [1], [2], [3], [4], [6], [7], [8], [9], [10], [11], [12], [13], [14], [15], [16], [17]. The split images are directed onto the single camera using a series of mirrors, which introduces minor parallax errors. These are offset by the drastic reduction in total system cost by requiring fewer camera/lens pairs. The resolution of the parallax error issue in a single-camera configuration has been demonstrated using advanced configurations requiring both beam-splitting optics and mirrors [4]. However, the introduction of parallax is particularly advantageous for tomography, which uses a series of two-dimensional images from different vantage points to reconstruct three-dimensional objects [1], [15]. Notably, a small parallax angle can even be useful for calculating three-dimensional flow structures whilst maintaining high-quality view multiplication [6]. The introduction of different optical paths allows each image to act as a distinct “simulated” camera image from a unique viewpoint, aiding significantly in the quality of three-dimensional reconstruction [1]. Even with access to multiple cameras, view-splitting is advantageous as it allows multiplying the effective number of available camera views [1], [3].

View splitting onto a single camera can be achieved using either a stereoscope configuration [13], [14], [15], [16], [17], [18], [19], a quadscope configuration [7], [8], [9], [10], [11], [12], or a fiber-based endoscope [20]. Stereoscopes typically use two sets of mirrors (or one set of mirrors and a beamsplitter) to cast image pairs onto the camera, while quadscopes use four sets to cast a series of four images on the camera. Commercially available stereoscopes (often denoted “image doubler” or “image splitter”) package the optical hardware within a self-contained unit, examples include: LaVision Image Doubler [21], Hamamatsu Photonics W-VIEW GEMINI Image Splitting Optics [22], and Cairn Research OptoSplit II [23]. While LaVision Image Doubler [21] uses a simple mirror or lens arrangement for advanced flow imaging with moderate object distances, advanced products such as Hamamatsu Photonics W-VIEW GEMINI Image Splitting Optics [22], and Cairn Research OptoSplit II [23] use multiple components such as beam splitters, dichroic mirrors, correction, and relay lenses, which are more suitable for high-resolution microscopic imaging with smaller object distances. These units are highly costly, starting near $10,000 (LaVision Image Doubler) and often greatly exceeding this mark. Commercial units are pre-calibrated, enclosed, and designed to optimize the imaged field-of-view, minimize cross-talk between image channels, and limit outside light contamination. Additionally, they also have coupling mechanisms to insert into the lens and camera systems, making them convenient for use. Of note, the commercially available image doublers often still require a posteriori image correction to account for optical distortions between the two view paths [13]. Quadscopes are generally not commercially available, with the notable exception of the Cairn Research MultiSplit V2 [24], which uses multiple wavelength/polarization-based beam splitters and filters to obtain four perfectly aligned multi-spectral views suitable for high-resolution microscopy. Low-cost devices for flow diagnostics applications or general imaging are not available, requiring researchers to construct custom-fabricated view splitters [7], [8], [9], [10], [11], [12]. The quadscope design is significantly more difficult to implement than the stereoscope design, which requires fitting and calibrating a double the number of optical components within the same limited space. As a result of design difficulty and the lack of available low-cost options, the availability and use of quadscopes are very limited at present.

Here, we describe a simple, low-cost, and open-source image view splitter (quadscope) designed for use in advanced flow diagnostics. The hardware is easy to build and integrates with standard optical components. All design files, build instructions, and details of the alignment procedure are included to facilitate the quick implementation of the system assembly. Image spatial resolution was computed to comprehend the effect of quadscope on image quality. Additionally, a focusability study was performed to characterize hardware performance with increasing object distances, quantifying changes in image map quality, parallax angles, pixel density, and size of the field-of-view (FOV). Validation was performed using the two-color pyrometry technique on a non-premixed methane/air flame (a practical thermofluidic system of interest). The quadscope successfully demonstrated the ability to perform simultaneous high-speed imaging and quantitative measurement. Compared to commercial hardware, the full field of view of each camera quadrant was not realized, and there was some image overlap. However, the huge cost reduction and open source nature of this design have the potential to accelerate the pace of research within the broader diagnostics community and democratize access to advanced diagnostic capabilities. Although the developed hardware is most suited for general imaging at moderate object distances, alternate designs with applications in high-resolution microscopy will be part of a future study.

## 2 Hardware description

The low-cost camera view splitter (quadscope) described in the present work provides up to four views of the target, for view multiplication with minimal parallax (for multivariate imaging) or views at multiple angles or orientations (for tomography). The assembly was designed to be built using open-source optical mounts (view splitter and guiding mirrors) and commercially available mirrors and optical components. The optical holders (view splitter and guiding mirror) are intended to be 3D printed using the provided files in the repository. The design allows quick mounting and demounting of the mirrors, making it convenient to replace optical components in the event of damage. In addition to the quadscope, an optional filter mount design is provided for multi-spectral imaging when used with the quadscope. The designed quadscope unit has the following features:

• Low-cost and open-source camera view splitter design for view multiplication with minimal parallax (for multivariate imaging) or user-set angles/orientations (for tomographic imaging).
• Compatible with commercially available optical components.
• Quick and easy mounting and demounting of the optical components.
• Option for editing/modifying the source CAD files according to the user’s needs.
• Optional dual-filter mount design for multi-spectral imaging when used with the quadscope.

## 3 Design files summary

The design files used to fabricate the quadscope system are summarized in Table 1. The files can be found in a data repository which can be accessed using the links found in Table 1.

1. Two different designs are provided for the quadscope: camera view splitter.
  (a) Quadscope_ViewSplitter_Type_01.SLDPRT - This quadscope design, once 3D printed, will house four broadband mirrors and can be used to generate four split views with minimal parallax angles between the views. This is ideal for multivariate imaging, where replicating the target image without significant optical distortion is required. This design is similar to the “stacked image doubler” configuration [6].
  (b) Quadscope_ViewSplitter_Type_02_Tomography.SLDPRT - This quadscope design, once 3D printed, will house four broadband mirrors and can be used to obtain four split views with user-controlled viewing angles (the parallax will be significant). This is ideal for tomographic or 3D imaging, where viewing the target from multiple angles enables effective 3D image reconstruction.
2. Quadscope_GuidingMirror_Adapter.SLDPRT - This mirror holder, once 3D printed, will house a broadband mirror and can be used to redirect the light coming from the target to the respective channels of the quadscope view splitter. Four of these are needed for four channels of the quadscope view splitter.
3. An optional spectral filter holder is designed, which is capable of housing two 50 mm diameter (thickness approx. 5 mm) spectral filters, allowing multi-spectral imaging of the target when used along with the quadscope assembly (View splitter and guiding mirrors assembly).
  (a) Spectral_Filter_Holder_Back_Plate.SLDPRT - This houses two spectral 50 mm filters.
  (b) Spectral_Filter_Holder_Front_Plate.SLDPRT - This, along with the spectral filter holder back plate, securely grips the filters.

**Table 1:** Design file summary.

Design filename	File type	Open source license	Location of the file
Quadscope_ViewSplitter_ Type_01.SLDPRT	CAD files (SolidWorks and STL)	CC BY 4.0	Repository link

Quadscope_ViewSplitter_ Type_02_Tomography.SLDPRT	CAD files (SolidWorks and STL)	CC BY 4.0	Repository link

Quadscope_GuidingMirror_ Adapter.SLDPRT	CAD files (SolidWorks and STL)	CC BY 4.0	Repository link

Spectral_Filter_Holder_ Front_Plate.SLDPRT	CAD files (SolidWorks and STL)	CC BY 4.0	Repository link

Spectral_Filter_Holder_ Back_Plate.SLDPRT	CAD files (SolidWorks and STL)	CC BY 4.0	Repository link

## 4 Bill of materials summary

The bill of materials used to fabricate the quadscope system is summarized in Table 2. The total for the bill of materials sums up to $1,208 ($2,678 including spectral filters). As the broadband silver mirrors (8×) are the major contributor to the bill of materials, alternative options from Edmund Optics (25 × 35 mm silver mirror, 4-6λ, Stock #89-484, Link) is provided for ease of procurement. However, the thickness of this mirror is 3 mm compared to 1 mm in the case of Thorlabs. Nonetheless, minor design modifications to the given SolidWorks part files can accommodate housing of the above mirror. Additionally, for general-purpose optics-related products such as posts, post holders, right-angle clamps, base plates, and mirror holders, both Edmund Optics and Newport Corporation vendors can serve as alternative sources. Bandpass filters can also be sourced from Thorlabs or Edmund Optics and nuts and screws can be sourced from Amazon.

**Table 2:** Bill of materials summary.

Designator	Component	Number	Cost per unit - USD $	Total cost - USD $	Source of materials	Material type
Quadscope: View Splitter	Broadband silver mirror	4	$103.91	$415.64	Thorlabs (Link)	Metal and glass

Quadscope: View Splitter	Mirror holder	1	$10	$10	3D printed	Polymer

Quadscope: Guiding Mirrors	Broadband silver mirror	4	$103.91	$415.64	Thorlabs (Link)	Metal and glass

Quadscope: Guiding Mirrors	Rectangular mirror holder	4	$5	$20	3D printed	Polymer

Quadscope - Hardware	Set Screw 8-32 Nylon Tip	1 (Package of 10)	$9.73	$9.73	McMaster-Carr (Link)	Metal and Polymer

Quadscope - Optics Hardware	1/2“ optical posts (3” length)	1 (Package of 5)	$29.64	$29.64	Thorlabs (Link)	Metal

Quadscope - Optics Hardware	1/2“ optical posts (6” length)	1 (Package of 5)	$38.92	$38.92	Thorlabs (Link)	Metal

Quadscope - Optics Hardware	1/2“ post holder (2” length)	1 (Package of 5)	$45.66	$45.66	Thorlabs (Link)	Metal

Quadscope - Optics Hardware	1/2“ post holder (3” length)	2	$10.29	$20.58	Thorlabs (Link)	Metal

Quadscope - Optics Hardware	Right angle clamp for 1/2” posts	2	$11.58	$23.16	Thorlabs (Link)	Metal

Quadscope - Optics Hardware	1” kinematic mirror holder	4	$44.78	$179.12	Thorlabs (Link)	Metal

Spectral Filter Assembly	10 nm line filters (550, 600, 700, & 750 nm)	4	$358	$1432	Andover Corporation (Link)	Glass and Polymer

Spectral Filter Assembly	Filter holder: front/back plates	2	$10	$20	3D printed	Polymer

Spectral Filter Assembly - Hardware	M4 screws and nuts	1 (package of 100	$18.10	$18.10	Mcmaster-Carr (Link1, Link2)	Metal

## 5 Build instructions

Building the quadscope view splitter requires the fabrication of two main components: (1) quadscope view splitter and (2) quadscope guiding mirrors. The build instructions additionally include the optional fabrication of a spectral filter housing.

### 5.1 Quadscope view splitter

**Fig. 1:**
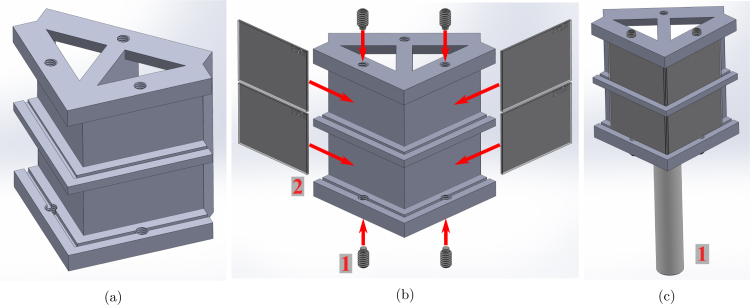
Quadscope view splitter assembly with instructions (for view multiplication with minimum parallax): (a) mirror holder (3D printed), (b) making of the view splitter assembly with steps (1 - 8-32 nylon-tip set screws, and 2 - Broadband silver mirrors), and (c) final view splitter assembly (1 - 1/2” optical post).

**Fig. 2:**
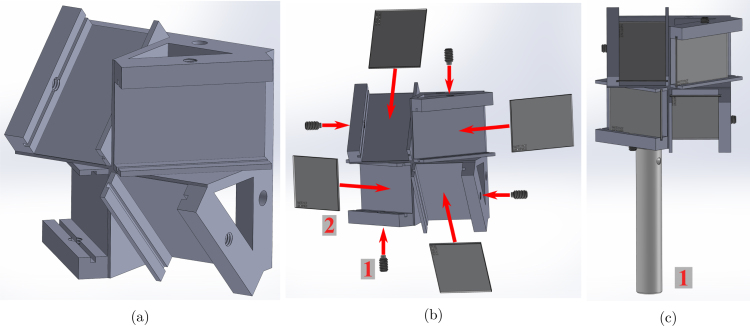
Quadscope view splitter assembly with instructions (for tomography): (a) mirror holder (3D printed), (b) making of the view splitter assembly with steps (1 - 8-32 nylon-tip set screws, and 2 - Broadband silver mirrors), and (c) final view splitter assembly (1 - 1/2” optical post).

The quadscope view splitter mirror holder is modeled in SolidWorks (2022), with two designs for minimizing parallax error (Quadscope_ViewSplitter_Type_01.SLDPRT) and tomographic application

(Quadscope_ViewSplitter_Type_02_Tomography.SLDPRT). The 3D-printed mirror holder contains four identical slots (at an angle of 45°) for placing the broadband silver mirrors (commercially available). Use the 8–32 nylon-tip set screws to secure the silver mirrors in place in their respective slots (nylon toward the mirror to prevent damage). Take caution while using the set screws, as over-tightening can lead to cracks in the thin mirrors, even if the nylon ends are soft to prevent damage to the mirrors. The best practice is to start tightening in the steps of half turns and decrease it to quarter turns or even one eighth of a turn as you continuously check the movement of the mirror in the slot. Following mounting of the mirrors, mount the quadscope view splitter onto a 1/2” (12.5 mm) optical post using the 8–32 set screws. A visualization of the build instructions for the quadscope view splitter assemblies is provided in Fig. 1, Fig. 2.

### 5.2 Quadscope guiding mirror

**Fig. 3:**
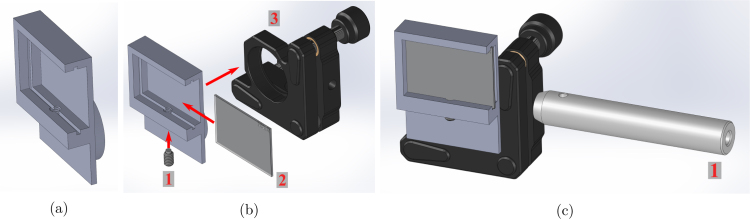
Quadscope guiding mirror assembly with instructions: (a) rectangular mirror holder (3D printed), (b) making of the guiding mirror assembly with steps (1 - 8-32 nylon-tip set screw, 2 - Broadband silver mirror, and 3 - 1” kinematic mirror holder), and (c) final guiding mirror assembly (1 - 1/2” optical post).

The quadscope guiding mirror holder is modeled in SolidWorks (Quadscope_GuidingMirror_Adapter.SLDPRT). The 3D-printed guiding mirror holder contains one slot for placing a broadband silver mirror. Use the 8–32 nylon-tip set screws to secure the silver mirrors in place in their respective slots (nylon toward the mirror to prevent damage). Mount the guiding mirror into the 1” (25 mm) diameter kinematic mirror holder with the bottom surface of the guiding mirror adapter roughly aligned with the bottom surface of the kinematic mirror holder shown in Fig. 3(b). Lastly, mount the kinematic mirror holder with the guiding mirror to the 1/2” (12.5 mm) optical post using the 8–32 set screws. Repeat this assembly for each of the four guiding mirror holders (for four channels of the quadscope view splitter). A visualization of the build instructions for the quadscope guiding mirror assembly is provided in Fig. 3.

### 5.3 Spectral filter (optional)

**Fig. 4:**
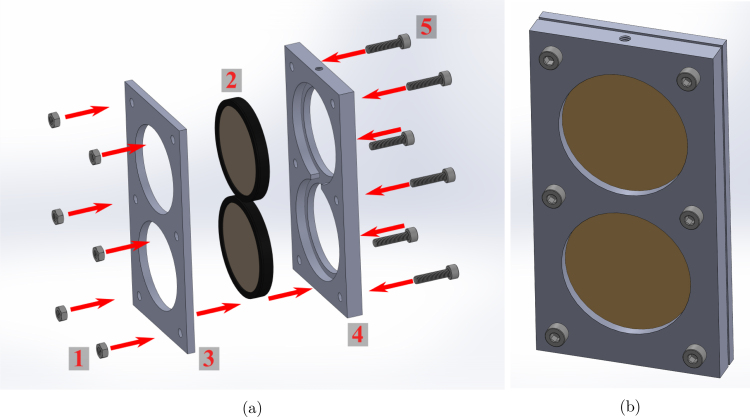
Spectral filter assembly with instructions: (a) making of the spectral filter assembly with steps (1 - M4 nuts, 2–50 mm diameter spectral filters, 3 - spectral filter holder front plate, 4 - spectral filter holder back plate, and 5 - M4 screws), and (b) final spectral filter assembly.

The spectral filter holder is modeled in SolidWorks (2022), with two files, one for the front plate

(Spectral_Filter_Holder_Front_Plate.SLDPRT) and the other for the back plate

(Spectral_Filter_Holder_Back_Plate.SLDPRT). The 3D-printed spectral filter holders contain two recesses for placing two 50 mm diameter (thickness ≈ 5 mm) spectral filters. Use M4 screws and nuts to secure the spectral filters in place in their respective recesses (ensure both filters are oriented in the matching direction). Mount the spectral filter assembly on a 1/2” (12.5 mm) optical post using the 8–32 set screws. Repeat this assembly for each of the two spectral filter holders. A visualization of the build instructions for the spectral filter assembly is provided in Fig. 4.

## 6 Operation instructions

First, the building and alignment process for the quadscope assembly (view splitter, four guiding mirrors, and optional two spectral filters) is provided. Following this, procedures for correcting the optical distortions for the quadscope views (image mapping) are detailed.

**Fig. 5:**
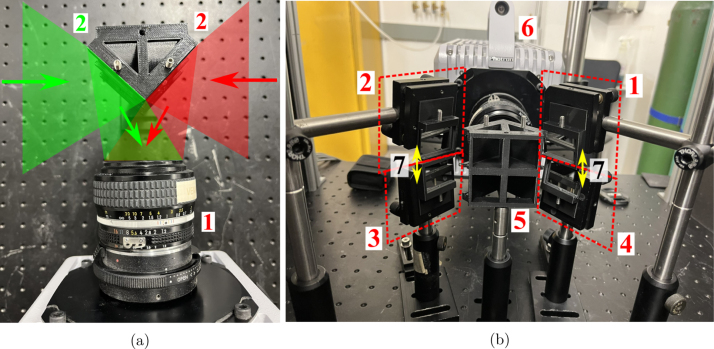
High-speed camera with the quadscope assembly: (a) quadscope view splitter (1 - Nikon 50 mm f/1.2 camera lens, 2 - Light processed by the view splitter), and (b) quadscope view splitter and guiding mirrors assembly (1–4 - Quadscope guiding mirrors, 5 - Quadscope view splitter, 6 - High-speed camera, and 7 - Vertical distance of 30 mm between the centers of the guiding mirrors).

### 6.1 Quadscope assembly and alignment process

**Fig. 6:**
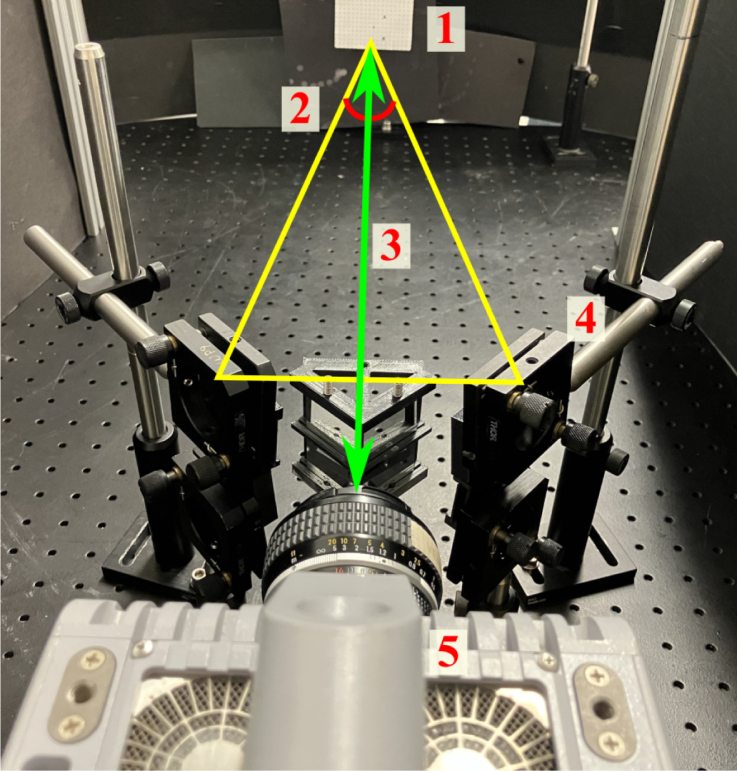
Image showing high-speed camera and quadscope assembly with a 90 mm × 90 mm dot grid kept at 850 mm from the camera (U). This dot grid is later used for distortion corrections of quadscope views (image mapping). The parallax angle between left and right guiding mirror views is also shown. The labels in the figure are as follows: 1–90 mm × 90 mm dot grid, 2 - Parallax angle (L/R), 3 - Object distance (U), 4 - Quadscope assembly, and 5 - High-speed camera.

**Fig. 7:**
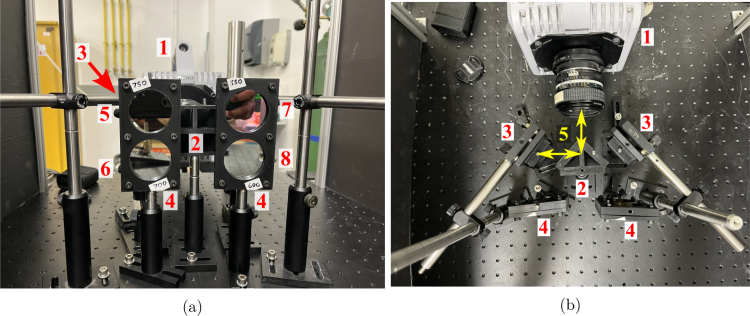
High-speed camera with the quadscope assembly for flame pyrometry: (a) view 1 (1 - High-speed camera, 2 - Quadscope view splitter, 3 - Quadscope guiding mirrors, 4 - Spectral filter assembly for pyrometry, and 5 to 8 - Bandpass filters of wavelengths 750 nm, 700 nm, 550 nm, and 600 nm respectively (FWHM - 10 nm), and (b) view 2 (1 - High-speed camera, 2 - Quadscope view splitter, 3 - Quadscope guiding mirrors, 4 - Spectral filter assembly for pyrometry, and 5 - Distance of 50 mm from the camera lens to the view splitter and from the view splitter to the guiding mirrors).

**Fig. 8:**
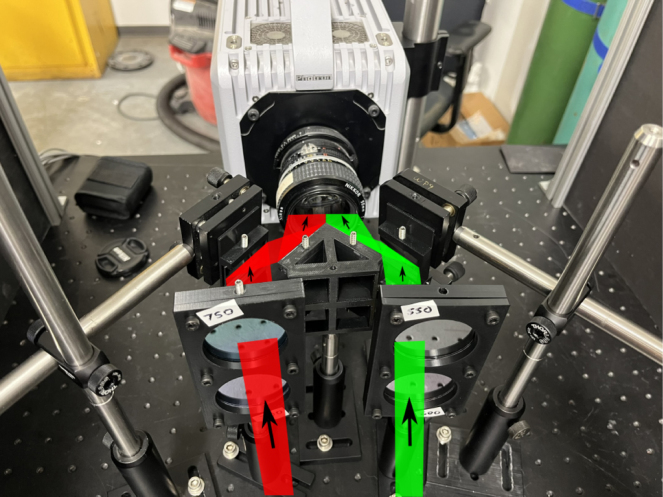
Splitting of light beam to create four views. The light beam is also spectrally separated with the use of appropriate filters.

**Fig. 9:**
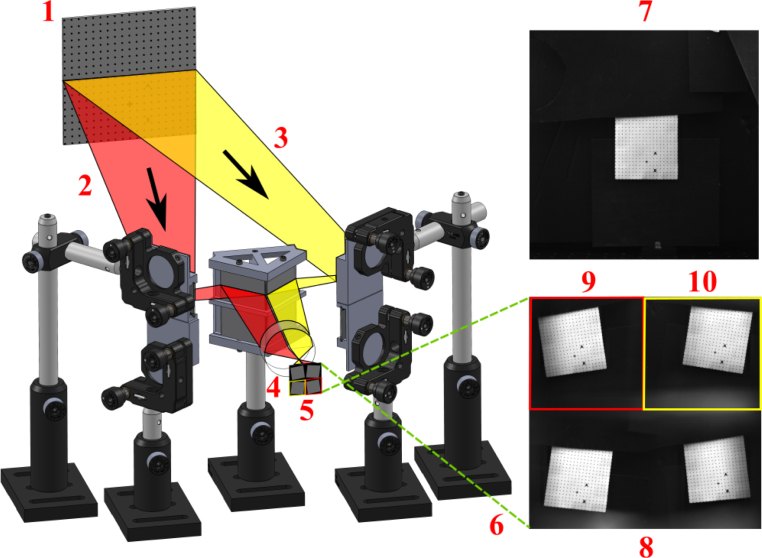
Optical ray paths from object plane to image plane for two sample quadscope views (Upper left view in red and upper right view in yellow). The labels in the figure are as follows: 1–90 mm × 90 mm dot grid (Object plane), 2 - Ray path for upper left view, 3 - Ray path for upper right view, 4 - Camera lens, 5 - Camera sensor (Image plane), 6 - Automatic lateral and vertical inversion by the camera electronics, 7 - Image without quadscope, 8 - Image with quadscope, 9 - Upper left view, and 10 - Upper right view.

**Fig. 10:**
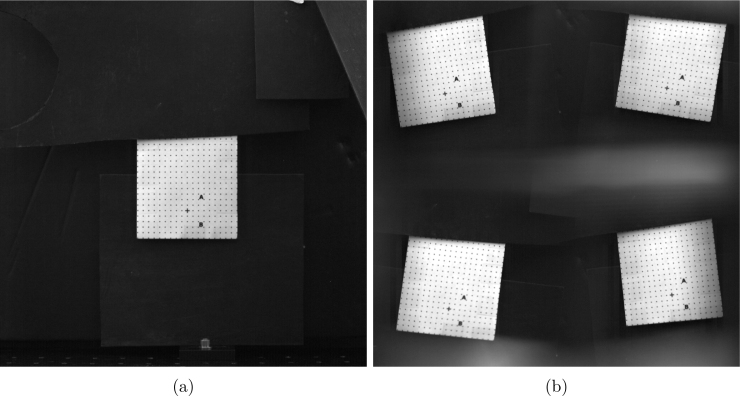
Image of a 90 mm × 90 mm (distance between the dots = 5 mm) dot grid: (a) single view without quadscope (Pixel density = 312.5μm, FOV = 320 mm × 320 mm), and (b) four split views with quadscope (Pixel density = 333.3μm, each quadrant FOV = 171 mm × 171 mm).

1. Place the quadscope view splitter centrally (vertically and horizontally) ≈ 50 mm away from the camera lens (50 mm f/1.2 lens used here). The location of the quadscope view splitter is determined by where all the light from the background is completely blocked, allowing only light from the side channels to enter the camera lens. This is shown in Fig. 5a (red and green shaded regions showing light from side channels). Starting with a larger distance from the camera lens where the background light is clearly visible, move the view splitter slowly toward the camera lens, marking the suitable location where the light behind the view splitter is barely visible. This is done by placing a white paper behind the view splitter, making the background distinctly bright (clearly visible when the quadscope view splitter is placed far away from the lens). Note that the location of the quadscope view splitter will vary with the focal length or viewing angle of the lens. For higher focal length lenses, the distance of the quadscope view splitter from the camera lenses will be higher due to the narrower field of view of these lenses.
2. Mount the guiding mirrors on the optical posts and post holders shown in Fig. 5b. The guiding mirrors should be placed at an angle of ≈ 45° to the viewing direction. The top and bottom guiding mirrors should be slightly tilted down and up, respectively, to look at the same target. Fig. 6 shows a 90 mm × 90 mm dot grid as the target placed 850 mm from the camera lens (defined as object distance U). With the above set coarse angles of the guiding mirrors, perform the final alignment by carefully controlling the horizontal and vertical tilt angles of the mirrors using the knobs in the corresponding kinematic mirror holders. Fig. 10b shows a sample image of the target dot grid after quadscope alignment. Optimal alignment is achieved by placing the image of the target in its respective quadrant FOV away from the center to limit crosstalk between quadscope views. Due to the placement of the guiding mirrors at an off-centered location, thereby a slight tilt in the viewing direction (parallax shown in Fig. 6), the images of the target might look defocused at larger apertures (shallow depth of field). Therefore, an appropriate aperture (f/4 in the present work) is chosen to balance the incoming light (lower amounts of light at lower apertures) and good focusability in the desired field-of-view. The images of the target might also look defocused due to differences in the path lengths between various quadscope views. This can be resolved by approximately matching the path lengths during the alignment process. Minor differences in path lengths can be accommodated with a smaller lens aperture (f/4 in the present work) by increasing the imaging depth of field.
3. (Optional) With the quadscope alignment completed, mount the two spectral filter assemblies on optical posts and post holders and place them in front of the guiding mirrors shown in Fig. 7. This step is required if the user wishes to perform multi-spectral imaging, e.g., flame pyrometry experiments performed as a part of validation and characterization in the current study. Various filters were chosen (750 nm, 700 nm, 600 nm, and 550 nm) for the pyrometry study shown in Fig. 7a. The choice of filters can vary with the intended application. Fig. 8 shows the light path from the target as it is processed by various filters, guiding mirrors, and channels of the view splitter to generate the split views in the camera. The horizontal location, vertical location, and orientation of each filter assembly were set in the present work by imaging a sooty ${\mathrm{CH}}_{4}$/air non-premixed flame detailed in Section 7.3. The position of the filter assembly is set where the flame appeared relatively bright in all four quadscope views (all four spectral regimes used in the present study), making sure the filter assembly does not block the light path for each view. Fig. 9 shows the optical ray paths from the object plane (90 mm × 90 mm dot grid) to the image plane (camera sensor size of 21 mm × 21 mm) as processed by two quadscope guiding mirrors (out of four in total), the quadscope view splitter, and the camera lens. With the red shaded region showing the ray path for the upper left (UL) quadscope view, the yellow shaded region shows the ray path for the upper right (UR) view. Although the actual ray path is 3D, sample 2D ray paths starting from the mid horizontal line of the 90 mm × 90 mm dot grid (object plane) are shown here for visual clarity. The image formed on the camera sensor is typically laterally and vertically inverted by the camera electronics (images shown on the right side of Fig. 9) to match the object plane orientation.

### 6.2 View distortion correction/image mapping

Figs. 10a and 10b show the image of the dot grid without and with the quadscope assembly, respectively. The images were captured using a monochrome CMOS high-speed camera (Photron, model: FASTCAM SA-X2) fitted with a Nikon Nikkor 50 mm f/1.2 lens at an aperture setting of f/4. The pixel density calculated for the image without the quadscope was 312.5μm, resulting in a field-of-view of 320 mm × 320 mm. With the placement of the quadscope, the path length to the target slightly increased, resulting in a pixel density of 333.3μm and a field-of-view of each quadrant to be 171 mm × 171 mm. Thus, the use of the quadscope multiplies the views of the target in the camera at the expense of diminishing the camera’s field of view. In addition, the multiple views in the camera appeared to be distorted (tilt angle of $6-7^{\circ }$) due to the unique path of the light beam as processed by each channel of the quadscope. Therefore, corrections for these image distortions or image mapping are necessary prior to pixel-to-pixel data comparison and processing from the multitude of camera views.

**Fig. 11:**
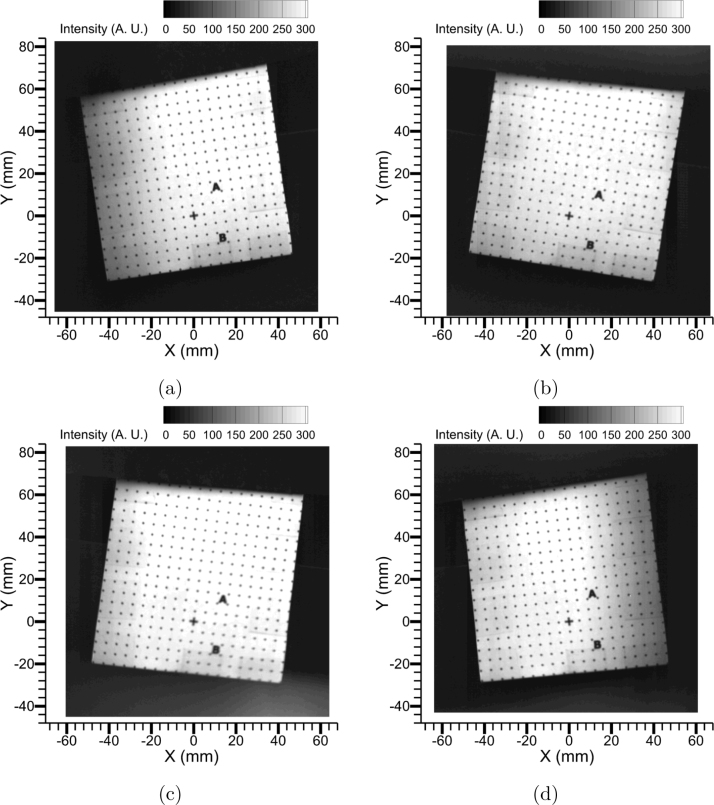
Original quadscope views cropped from Fig. 10b: (a) view 1 (Upper left), (b) view 2 (Upper right), (c) View 4 (Lower left), and (d) View 3 (Lower right).

**Fig. 12:**
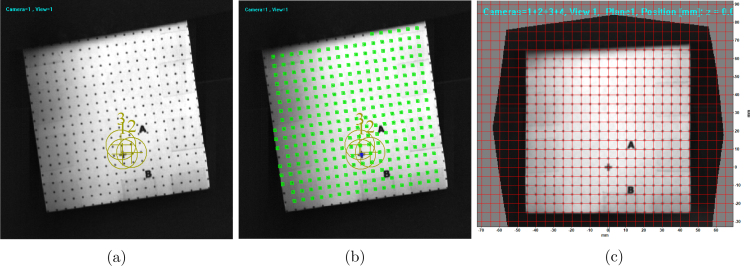
Image mapping process for the distorted quadscope views: (a) step 1 - marking reference locations in all quadscope views, (b) step 2 - automatic grid point detection by the software, and (c) Step 3 - overlaid mapped quadscope views showing good fit quality.

**Fig. 13:**
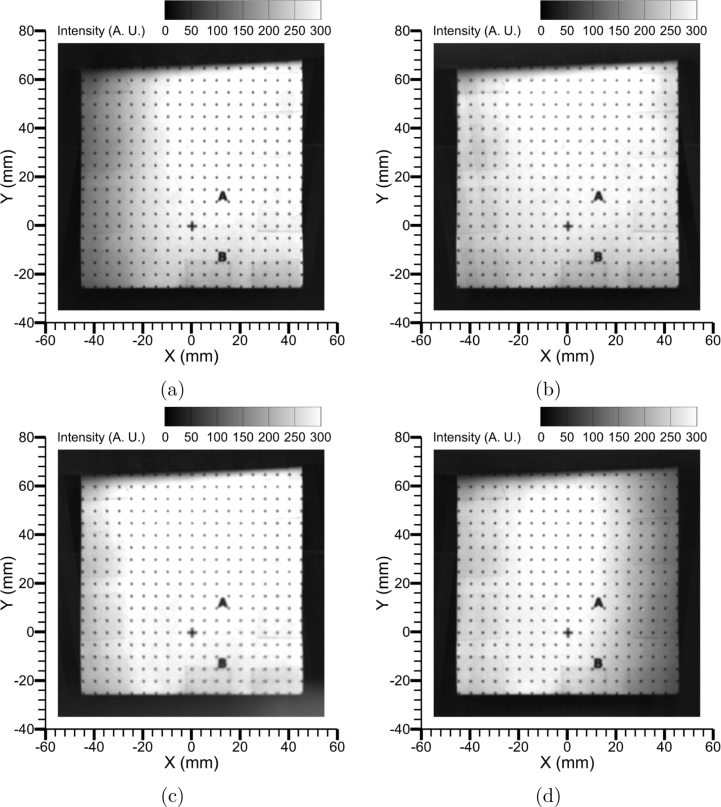
Corrected quadscope views (cropped appropriately) after image mapping: (a) view 1 (Upper left), r.m.s fit = 0.148, (b) view 2 (Upper right), r.m.s fit = 0.167, (c) view 4 (Lower left), r.m.s fit = 0.158, and (d) view 3 (Lower right), r.m.s fit = 0.157.

Before the start of the image mapping process, the four views captured in the compound camera image (Fig. 10b) were cropped to create independent datasets (Fig. 11a-d). The image mapping process involves a few ubiquitous steps shown in Fig. 12 regardless of the software used for processing. For the present work, DaVis 8.3 [25] software is used for image mapping with MATLAB or the open-source Calibration Visualizer (CalVi) [26], [27] software in Python being alternative options. The calibration process starts with detailing a few specifications, such as the number of views (4), coordinate systems (1), diameter (1 mm), spacing of the dots (5 mm) in the grid, etc. Following this, the user marks a few reference locations (three or more dots in the grid) in all four views (see Fig. 11). A sample image (View 1: upper left) with reference location markings is shown in Fig. 12a. With the reference locations marked in all views and the specifications of the dot grid, the software detects all the grid points automatically, marked in green and blue squares in Fig. 12b. Here, the blue square is considered as origin in all views, which was marked by the user in step 1. After the detection of all grid points in step 2, the software performs image mapping based on various fitting algorithms such as the camera pin-hole model or the 3rd order polynomial model, etc [26]. Fig. 12c shows the overlay of all corrected quadscope views following the image mapping procedure using the 3rd order polynomial fitting algorithm. The figure shows excellent overlap of all quadscope views with an average r.m.s. fitting error computed to be 0.158. A lower r.m.s. fitting error (closer to 0 and away from 1) suggests better distortion correction by the image mapping software, leading to perfect overlap of all quadscope views on top of each other. Fig. 13a-d shows the corrected quadscope views of each channel after image mapping. This developed calibration mapping is also used to correct quadscope views of other image sets, e.g., flame luminosity images reported in Section 7.4.

## 7 Validation and characterization

A set of validating experiments was conducted to test this hardware. First, the image quality of various quadscope views was obtained by computing the spatial resolution and later compared with images captured without the quadscope to comprehend the changes made by the hardware. Following this, a focusability study was performed to characterize the hardware capability with increasing object distance, with emphasis on pixel density and field-of-view. Subsequently, the quadscope was used to image flame luminosity from a methane/air non-premixed flame to demonstrate simultaneous imaging on a real thermofluidic flow of interest. Spectral filters were added to the quadscope assembly to perform multispectral measurements of soot radiation. Finally, flame temperature using two-color pyrometry was calculated. The hardware is not compared with the commercial product (Cairn Research MultiSplit V2 [24]) due to the high cost associated with procuring the product.

### 7.1 Image quality: spatial resolution

To comprehend the changes in image quality due to the quadscope assembly, spatial resolution for each quadscope view is calculated and compared with the spatial resolution achieved without the use of the quadscope. This is achieved by capturing images of a sharp knife edge (bright color) in a dark background, and fitting an error function to the light intensity captured by the camera [28], [29]. With a step function in reality (highly resolved), the image in the camera often looks smeared (fitted by an error function) due to a finite spatial resolution. The gradient of this error function forms a Gaussian curve, and the spatial resolution is denoted as the linewidth (Full width at half maximum or FWHM) of the curve. Spatial resolution in pixel units was converted to μm using the values of pixel density. Table 3 reports the pixel densities and the calculated spatial resolutions for the original view without the quadscope and four split views with the quadscope. The increase in pixel density (6.7%) from 312.5μm to 333.3μm with the use of quadscope is due to a slight increase in the path length reported in Section 6.2. The minor differences in path lengths for various quadscope views are observed to be insensitive to the pixel density, keeping the value unchanged. On the contrary, the spatial resolution changed between 890.4μm and 970.9μm for various quadscope views. The spatial resolutions obtained with the quadscope show a 39%–51% decrease (higher values mean decreased spatial resolutions) compared to the images captured without the quadscope (640.8μm). This is most likely due to minor path length differences, parallax angles, and the focusability effect. Additional design changes to improve the spatial resolution with the use of quadscope will be part of a future study.

**Table 3:** Summary of image quality parameters with and without quadscope.

Quadscope use	View number	Pixel density	Spatial resolution	Change %
No	–	312.5 μm	640.8 μm	–
Yes	1 (Upper Left)	333.3 μm	890.4 μm	39
Yes	2 (Upper Right)	333.3 μm	936.3 μm	46
Yes	3 (Bottom Right)	333.3 μm	970.9 μm	51.5
Yes	4 (Bottom Left)	333.3 μm	923.5 μm	44

**Table 4:** Summary of quadscope focusability study with increasing object distance.

Object distance (U)	Pixel density	Quadrant FOV	Parallax angle (L/R) (α)	Parallax angle (T/B) (β)	Average r.m.s. fit
850 mm	333.33 μm	171 mm ×171 mm	$7^{\circ }$±$0.7^{\circ }$	$2.1^{\circ }$±$0.2^{\circ }$	0.158
775 mm	312.5 μm	160 mm ×160 mm	$7.8^{\circ }$±$0.8^{\circ }$	$2.4^{\circ }$±$0.3^{\circ }$	0.163
700 mm	294.1 μm	151 mm ×151 mm	$8.7^{\circ }$±$0.86^{\circ }$	$2.6^{\circ }$±$0.3^{\circ }$	0.190
625 mm	250 μm	128 mm ×128 mm	$9.8^{\circ }$±$0.98^{\circ }$	$2.9^{\circ }$±$0.4^{\circ }$	0.206
550 mm	217.4 μm	111 mm ×111 mm	$11.2^{\circ }$±$1.2^{\circ }$	$3.4^{\circ }$±$0.4^{\circ }$	0.238
475 mm	192.3 μm	98 mm ×98 mm	$13.2^{\circ }$±$1.3^{\circ }$	$4.0^{\circ }$±$0.5^{\circ }$	0.258

### 7.2 Focusability study

**Fig. 14:**
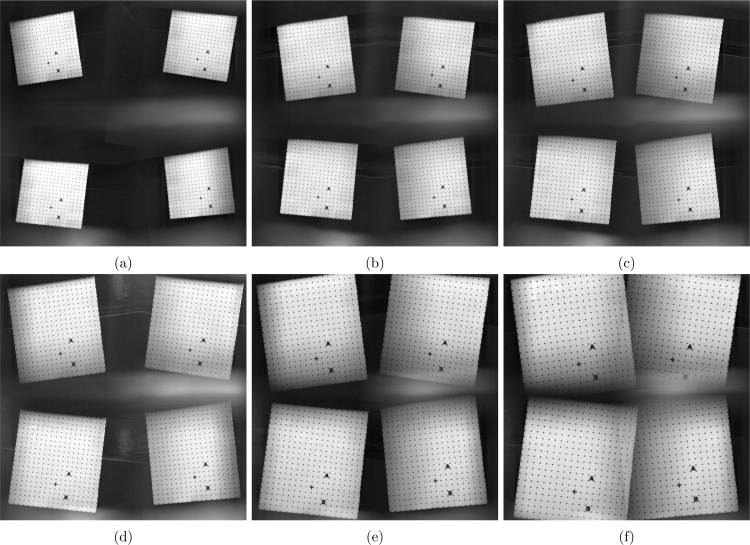
Split views of the 90 mm × 90 mm dot grid with the quadscope at various object distances (U) from the camera: (a) U = 850 mm (Pixel density = 333.3μm), (b) U = 775 mm (Pixel density = 312.5μm), (c) U = 700 mm (Pixel density = 294.1μm), (d) U = 625 mm (Pixel density = 250μm), (e) U = 550 mm (Pixel density = 217.4μm), and (f) U = 475 mm (Pixel density = 192.3μm).

To understand the effects of object distances (U) on image focusability and thereby the quality of the image mapping process, a series of experiments was performed by imaging the dot grid at object distances (U) from 850 mm to 475 mm in decrements of 75 mm. Fig. 14a-f shows all quadscope views for the various object distances. As the U decreased, the calculated pixel densities decreased, making the images of the dot grid appear bigger, filling the field-of-view of each quadrant. The calculated pixel densities for each U, along with the field-of-view size of the quadrant, are reported in Fig. 14 and Table 4. Although visually the images appear to be in focus even at lower object distances, overlap and cross-talk between the views are apparent at U = 475 mm. Additionally, the parallax angles (left/right and top/bottom) would also be higher at lower U. The parallax angle between the left and right quadscope guiding mirror viewing orientations (Parallax Angle (L/R) or α shown in Fig. 6) is defined as follows:

(1) $$\alpha =2\;\tan^{-1}\left(\frac{50\;\pm 5\;}{U\;-50\;}\left[\mathrm{mm}\right]\right)$$

where 50 ± 5 mm is the distance between the center of the quadscope view splitter and guiding mirror (horizontal direction), U is the object distance (distance between the target and the camera lens), and 50 mm is also the distance between the camera lens and the quadscope view splitter. This is shown in Fig. 7b. Here, U - 50 mm is the distance between the target and the center of the quadscope view splitter.

**Fig. 15:**
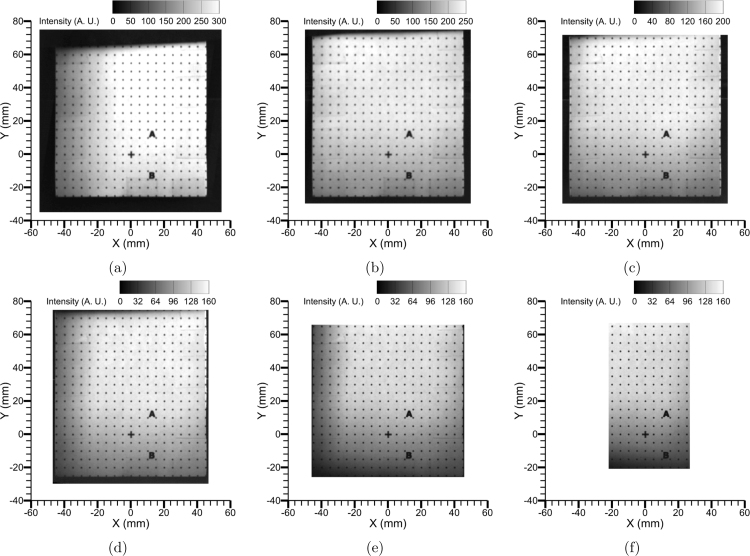
Corrected quadscope views (View 1: Upper left) for various object distances (U) from the camera: (a) U = 850 mm (r.m.s. fit = 0.148), (b) U = 775 mm (r.m.s. fit = 0.164), (c) U = 700 mm (r.m.s. fit = 0.186), (d) U = 625 mm (r.m.s. fit = 0.2), (e) U = 550 mm (r.m.s. fit = 0.23), and (f) U = 475 mm (r.m.s. fit = 0.256).

Similarly, the parallax angle between the top and bottom quadscope guiding mirror viewing orientations (Parallax Angle (T/B) or β) is defined as follows:

(2) $$\beta =2\;\tan^{-1}\left(\frac{15\;\pm 2\;}{U\;-50\;}\left[\mathrm{mm}\right]\right)$$

where 15 ± 2 mm is the distance between the center of the quadscope view splitter and guiding mirror (vertical direction, see Fig. 5b), and U - 50 mm is the distance between the target and the center of the quadscope view splitter or guiding mirror (distance between the view splitter and guiding mirror is much lower than the distance to the target). Table 4 reports the parallax angles α and β to vary between 7°-13° for left and right views and 2°-4° for top and bottom views, with higher parallax angles at lower U. Fig. 15 shows the final corrected views (only View 1: Upper left is shown, however, all the views are perfectly mapped as shown in Fig. 13) for all object distances. At lower U, the overlap between the channels leads to a smaller usable field-of-view, e.g., at U = 475 mm, the usable FOV is 48 mm × 84 mm compared to the quadrant FOV of 98 mm × 98 mm. Overall, a maximum usable FOV of 66% of the quadrant FOV is observed in this study, in contrast to a larger usable FOV % accessible with commercial designs [21], [22], [23], [24]. The remaining 33% of the quadrant FOV is used to accommodate image distortions, channel overlap, and crosstalk. To limit crosstalk and channel overlap, the imaging FOV is filled with a black background (done by using black cardboard). The usable area in each quadrant FOV can be improved by lowering the image distortion (lowering the current tilt angle of $5^{\circ }-7^{\circ }$ to nearly vertical or 0°), possibly with the view channels in the quadscope view splitter oriented 45° in both horizontal (for left and right views) and vertical (for top and bottom views) directions away from the center point. This alternative design to increase usable FOV % will be part of a future study.

Table 4 additionally reports the quality of the image mapping or distortion correction (fitting r.m.s.), which worsens at lower U again due to increased distortions, parallax angles, overlap, and crosstalk. For all the qualitative and quantitative studies performed in the subsequent sections (flame luminosity images and two-color pyrometry), an object distance of U = 850 mm was chosen to ensure minimum parallax angles, better image mapping, and importantly enough space between the views so that the circular filters can be placed in front of the quadscope without blocking any of the views. Without filters, the object distance could be reduced to U = 700 mm or 625 mm, thus increasing the image resolution with a decent parallax angle and image mapping quality. Alternatively, with higher focal length lenses, the image resolution can be enhanced without compromising on parallax angles or image mapping quality, although the amount of light collected by the system will be lower, and this will result in a dimmer image. Finally, an intricate balance between the image resolution, parallax angles, image mapping quality, and light collected by the system has to be performed to determine the optimal object distance (U).

### 7.3 Flame imaging and two-color pyrometry methodology

Validating experiments were performed to demonstrate simultaneous high-speed imaging with quantitative measurement in a real, practical thermofluidic flow of interest: a non-premixed methane flame. For quantitative measurement, the two-color pyrometry method [4], [30], [31], [32] was employed. This method relies on the spectral dependence of radiation according to Planck’s Law [31], [32]:

(3) $$B_{\lambda }\left(\lambda ,T\right)=\epsilon \frac{2hc^{2}}{\lambda^{5}}{\left[exp\left(\frac{hc}{\lambda k_{B}T}-1\right)\right]}^{-1}$$

where $B_{\lambda }$ is the spectral radiance, λ is the wavelength, T is the temperature, ϵ is the emissivity, h is the Planck constant, c is the speed of light, and $k_{B}$ is the Boltzmann constant. The temperature is determined based on the intensity ratio, $\phi_{r}$, of two colors:

(4) $$\phi_{r}=\frac{\Psi_{1}\int B\left(\lambda_{1},T\right)\chi_{1}\left(\lambda_{1}\right)d\lambda }{\Psi_{2}\int B\left(\lambda_{2},T\right)\chi_{2}\left(\lambda_{2}\right)d\lambda }$$

where $\lambda_{i}$ and $\chi_{i}$ are the selected color wavelength and corresponding spectral sensitivity of the bandpass filter. This work utilizes two bandpass filters centered at $\lambda_{1}$ = 750 nm and $\lambda_{2}$ = 700 nm, both with 10 nm full width at half maximum (FWHM). C = $\Psi_{1}/\Psi_{2}$ is a calibration factor that is required to correct for the absolute light intensity, lens optics, internal camera electronics, and wavelength sensitivity [32]. A blackbody radiator (Infrared Systems Development Co., model: IR-563/301) with a maximum temperature T = 1320 K and an accuracy of ± 1°K was used to obtain the calibration factor and verify camera linearity. A greybody assumption was used for the emissivity of soot. Notably, this assumption results in large temperature measurement uncertainties for this technique [33], as opposed to laser-based thermometry approaches [34], [35], [36].

**Fig. 16:**
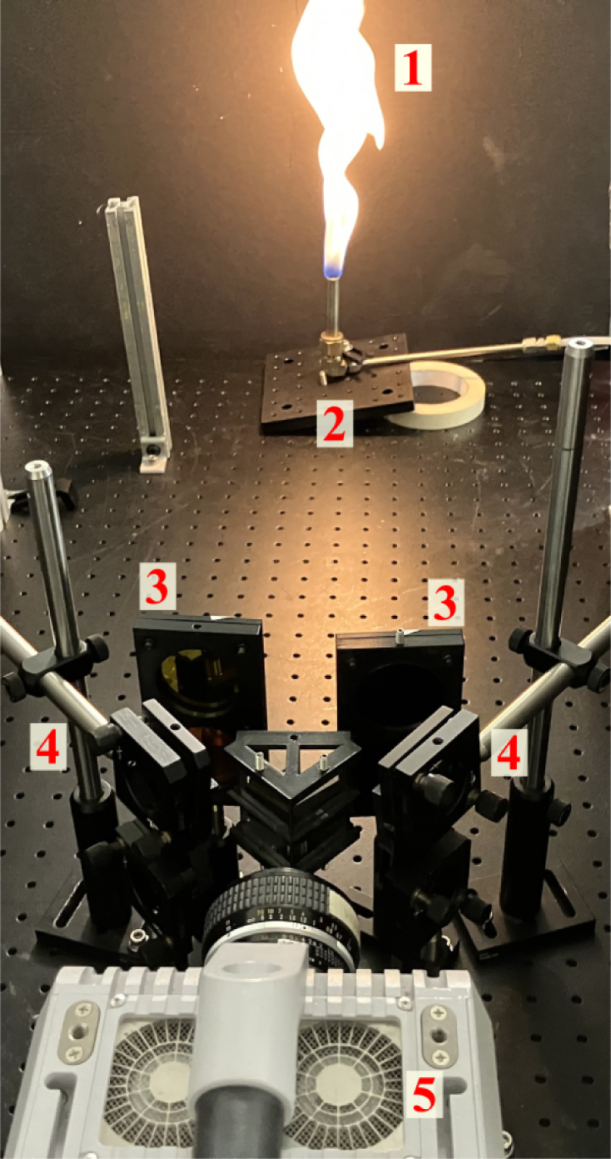
Flame pyrometry experimental setup with a sample ${\mathrm{CH}}_{4}$/air non-premixed flame. The labels are as follows: 1 - Soot, 2 - ${\mathrm{CH}}_{4}$/air non-premixed flame, 3 - Spectral filters for pyrometry, 4 - Quadscope assembly, and 5 - High-speed camera.

Fig. 16 shows an image of the flame pyrometry experimental setup with a sample ${\mathrm{CH}}_{4}$/air non-premixed flame. The methane jet was issued from a tube with a diameter of 12.7 mm at a flow rate of 3.5 SLPM into standard air. The center of the burner exit was positioned at an object distance of 850 mm. Images were captured using a monochrome CMOS camera (Photron, model: FASTCAM SA-X2) at 500 Hz, fitted with a Nikon Nikkor 50 mm f/1.2 lens with an aperture setting of f/4. Simultaneous high-speed imaging of the flame using all four views was first performed to demonstrate the capability for view multiplicity. Next, the spectral filter assembly was placed within the quadscope arrangement to perform the quantitative measurement of flame temperature using the two-color pyrometry technique.

### 7.4 Simultaneous high-speed imaging results

**Fig. 17:**
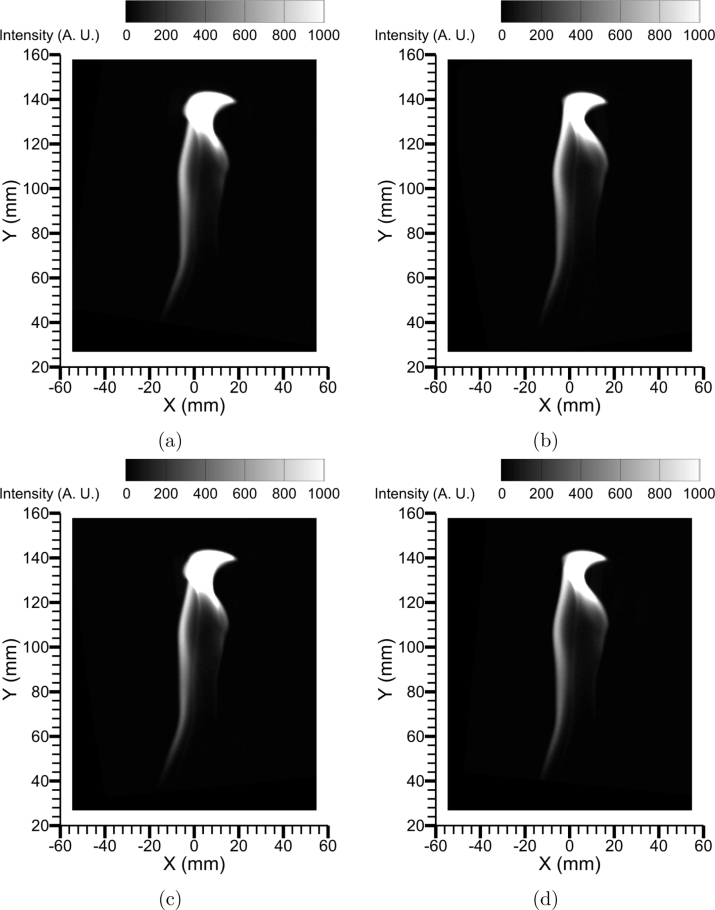
Corrected flame luminosity images from quadscope views following image mapping procedure: (a) view 1 (Upper left), (b) view 2 (Upper right), (c) view 4 (Lower left), and (d) view 3 (Lower right).

Fig. 17 shows the corrected flame luminosity images from the quadscope views following the image mapping procedure. Images were corrected using the known calibration (Section 7.2). The results affirm the hardware’s capability for high-speed view multiplication.

Minor parallax errors were observed between the four frames because the flame has a real finite thickness (in comparison to the flat calibration sheet). The parallax error is most relevant between the left- and right-hand side images, seen as the “thinning” of the flame on the right-hand side. Parallax error is also observed between the top and bottom frames, but this error is considerably less owing to the mirror arrangement. The optical path orientation is much different for the left- and right-hand side, whereas for the top and bottom mirrors, it is somewhat similar. The difference in viewing angle between the left- and right-hand sides (and resulting parallax error) decreases with increasing object distance; however, this must be appropriately balanced with increasing pixel density or decreasing image resolution. Of note, minor pixel shifting (≤ 2 pixels) using cross correlation as a target was required to perfectly align the flame views.

**Fig. 18:**
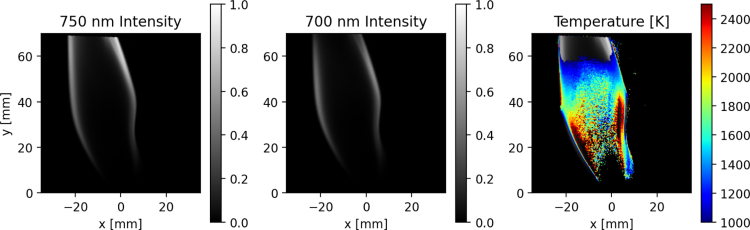
Flame irradiance at 750 and 700 nm along with the flame temperature obtained using the two-color pyrometry technique (See supplementary video 1)

**Fig. 19:**
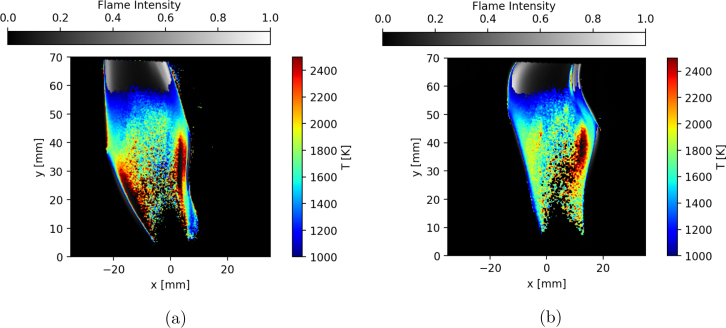
Instantaneous snapshots of flame temperature obtained using the two-color pyrometry technique: (a) image 1, and (b) image 2.

### 7.5 Quantitative temperature measurement results

Temperature measurement is performed by including the spectral filter holder in the quadscope arrangement and using the two-color pyrometry method. Fig. 18 shows the flame irradiance in the 750 and 700 nm along with the obtained instantaneous flame temperature. The 750 and 700 nm flame intensities align very well (low parallax error), allowing for more precise quantitative measurement. The ratio of intensity between both channels is used to calculate the temperature according to Eq. (4). The temperature is overlaid on the combined flame luminosity to provide context for the region in which the temperature is obtained. Temperature is only calculated at pixels in which the intensity of both the 750 nm and 700 nm channels is above a certain threshold, eliminating temperature calculation in the regions of lower signal-to-noise ratio (SNR).

Fig. 19 shows the instantaneous flame temperature for two instances in time using the two-color pyrometry technique, overlaid on top of the flame luminosity. The irradiance is collected at high speed, allowing for the instantaneous quantification of temperature. The maximum flame temperature measured is around 2400 K when averaged across the entire sampling duration. This agrees well with the calculated maximum adiabatic flame temperatures for methane in air, T=2233 K, calculated using the GRI-Mech 3.0 [37]. Actual instantaneous temperatures within the flame are expected to be higher (super-adiabatic temperatures) due to the complex chemical kinetics. This puts the measured temperature well within the expected bounds of uncertainty (∼5%) for this methodology [33].

### 7.6 Capability and limitations

A summary of the capabilities of this hardware assembly is as follows:

• Suitable for multivariate or multidimensional quantitative flow diagnostic measurements. Provides four views per camera.
• Includes two view splitter designs for use in 1) view multiplication with minimum parallax and (2) tomographic imaging.
• Fast assembly and easy integration with standard optical components.
• (Optional) integration of quadscope with spectral filtering included within the design.

and a summary of limitations is as follows:

• Minor parallax errors are unavoidable. Decreasing parallax error is observed with increasing object distance, which must be balanced with decreasing image resolution.
• Cannot access the full field-of-view in each camera quadrant. Limited by image overlap, crosstalk, and distortion.
• Set-up duration is longer than commercially available alternatives. (Balanced by large cost reduction)
• Decreasing calibration fit accuracy with increasing image resolution

### 7.7 Future works

Future work will involve addressing the current hardware limitations. Set-up duration and outside light contamination could be reduced by enclosing the quadscope with guide mirrors and making a standalone unit similar to the LaVision Image Doubler [21]. The unit could also have a coupling mechanism with the camera lens for easy installation. Improvements in the available FOV may be realized by:

• limiting cross-channel overlap by creating barriers between the quadscope view splitter mirrors
• Optimizing quadscope view splitter mirror angles and locations to “straighten” resulting image frames (will additionally improve calibration fit accuracy and increase usable pixels in each quadrant)
• Designing improved configurations [4] to further minimize parallax error or utilize beam-splitting optics.

The successful demonstration of the developed quadscope in view multiplication provides impetus to the authors’ ongoing efforts to develop economical diagnostic tools [38] for application in high-speed reacting flows of practical interest [39].

## 8 Conclusion

State-of-the-art flow diagnostics rely on the ability to perform multivariate and multidimensional measurements, allowing researchers to effectively study complex flow phenomena. Generating multiple views of a target onto a single camera using view splitting is an attractive means to reduce system cost and complexity; however, generating multiple views relies on expensive commercial image doubling lenses (∼$10,000) or custom-fabricated designs, hindering widespread use. This work describes the design and operation of a low-cost ($1,208) open-source image view splitter to service the advanced experimental flow diagnostics community.

A set of validating experiments was conducted to test this hardware. The system was characterized through a computation of image spatial resolution and a focusability study, quantifying the changes in parallax angles, image mapping quality, pixel density, and field-of-view size with increasing object distance. Experiments were then performed to demonstrate simultaneous high-speed imaging with quantitative measurement in a real, practical thermofluidic flow of interest: a non-premixed methane flame. The results demonstrated the successful operation of the hardware in collecting and aligning high-speed images. The quantified flame temperature from two-color pyrometry agreed well with calculated adiabatic flame temperatures. Although the current hardware is well-suited for general imaging at moderate object distances with demonstrated applications in flow diagnostics, alternative designs with applications in microscopy will be part of a future study.

## CRediT authorship contribution statement

**Abinash Sahoo:** Writing – review & editing, Writing – original draft, Visualization, Validation, Methodology, Investigation, Formal analysis, Conceptualization. **Ryan D. DeBoskey:** Writing – review & editing, Writing – original draft, Visualization, Validation, Methodology, Investigation, Formal analysis. **Venkateswaran Narayanaswamy:** Writing – review & editing, Supervision, Resources, Funding acquisition, Conceptualization.

## Ethics statement

The authors declare that they complied with the ethical guidelines of HardwareX and that they do not have any external influences that could have affected this work.

## Declaration of competing interest

The authors declare that they have no known competing financial interests or personal relationships that could have appeared to influence the work reported in this paper.

## Appendix A Supplementary data

Video S1 Flame irradiance at 750 and 700 nm along with the flame temperature obtained using the two-color pyrometry technique (Single frame in Fig. 18).
